# Integrative tumour mutation burden with CD39 and PD-L1 for the prediction of response to PD-L1 blockade and adjuvant chemotherapy in muscle-invasive bladder cancer patients

**DOI:** 10.1038/s41416-022-01943-y

**Published:** 2022-08-23

**Authors:** Chunnan Liu, Zhaopei Liu, Kaifeng Jin, Han Zeng, Fei Shao, Yuan Chang, Yiwei Wang, Le Xu, Zewei Wang, Yu Zhu, Weijuan Zhang

**Affiliations:** 1 Department of Urology, Fudan University Shanghai Cancer Center, Shanghai, China; 2 Department of Biochemistry and Molecular Biology, School of Basic Medical Sciences, Fudan University, Shanghai, China; 3 Department of Urology, Zhongshan Hospital, Fudan University, Shanghai, China; 4 Department of Oncology, Shanghai General Hospital, Shanghai Jiao Tong University School of Medicine, Shanghai, China; 5 Department of Urology, Shanghai Ninth People’s Hospital, Shanghai Jiao Tong University School of Medicine, Shanghai, China; 6 Department of Urology, Ruijin Hospital, Shanghai Jiao Tong University School of Medicine, Shanghai, China; 7 Department of Immunology, School of Basic Medical Sciences, Fudan University, Shanghai, China

**Keywords:** Bladder cancer, Cancer immunotherapy, Chemotherapy, Tumour immunology

## Abstract

**Background:**

CD39, a rate-limiting enzyme to convert extracellular ATP (eATP) to adenosine, has been reported to be a key modulator of immune response, but its correlation with therapeutic sensitivity remains obscure. We conducted this study to determine whether the integration of CD39 and traditional biomarkers could improve the prediction of responsiveness to PD-L1 blockade and platinum-based chemotherapy.

**Methods:**

We retrospectively enrolled a total of 760 patients from IMvigor210 trial, TCGA database and Zhongshan Hospital in this study. We constructed the CPT scoring system based on CD39, PD-L1 and tumour mutation burden (TMB) and validated its efficacy in predicting therapeutic responsiveness in MIBC patients. Kaplan–Meier survival and Cox regression analyses were applied to assess clinical outcomes of patients.

**Results:**

The CPT scoring system could predict the response to PD-L1 blockade and platinum-based chemotherapy. The CPT score was positively correlated with APOBEC mutational signature and SNV neoantigens enrichment, antigen presentation, and TCR signalling. High CPT score also indicated the inflamed immune phenotype and basal/squamous molecular subtype.

**Conclusions:**

CD39 expression is closely correlated with the immunogenic contexture of MIBC. Integrating CD39 with PD-L1 and TMB could stratify the sensitivity of patients with MIBC to PD-L1 blockade and platinum-based chemotherapy.

## Introduction

Muscle-invasive bladder cancer (MIBC) is one of the most lethal urinal malignancies, with a 5-year survival rate of <15% in patients who do not receive treatment [1]. Cisplatin-based adjuvant chemotherapy (ACT) has been the main strategy of systematic management of MIBC over the past decades [2]. Immune checkpoint inhibitors (ICIs) have revolutionised the therapeutic landscape with advanced development [3]. However, only a minority of patients could benefit from these treatments [4]. Thus, there is an urgent need to identify patients who will present a satisfying therapeutic response and to further achieve better clinical outcomes.

PD-L1 has been demonstrated to be an important biomarker for patient selection for ICIs, of which modest sensitivity is attracting increasing attention [5]. In addition, tumour mutation burden (TMB) has also been reported to be a useful biomarker for ICIs across cancer types such as melanoma and non-small cell lung cancer since it was observed that somatic mutations were key drivers in the generation of tumour-specific neoantigens [6]. However, clinical trials such as IMvigor210 and JAVELIN Bladder 100 trials indicated that single PD-L1 or single TMB could not ideally predict the responsiveness and they still missed large subgroups of patients who could potentially benefit from the therapy [7, 8]. Additionally, there has been a paucity of research regarding the combination of both biomarkers.

CD39, also called ectonucleoside triphosphate diphosphohydrolase 1, is one of the rate-limiting enzymes to convert extracellular ATP (eATP) to adenosine [9]. Within the tumour microenvironment (TME), the enrichment of eATP released from stressed or dying tumour cells could stimulate cell inflammation activity, including NLRP3 inflammasome activation, thus triggering the activation of T cells [10]. With a negative feedback mechanism, CD39 was upregulated under the stimulation of T cell receptor (TCR) to suppress activated immune response [11]. Thus, CD39 is a biomarker of tumour-reactive T cells and a potential indicator for an inflamed TME, which makes it a possible therapeutic biomarker in MIBC [12, 13]. Extensive research has shown that biomarkers characterising an inflamed TME could function as complementary predictors of therapeutic response independent of PD-L1 and TMB [14, 15].

Therefore, we cautiously hypothesised that the responsiveness of the systemic treatment in MIBC patients could be better predicted with the combination of CD39, PD-L1 and TMB. In the current study, we proposed a “CPT score”, which was derived by incorporating CD39 expression, PD-L1 expression and TMB level to evaluate the responsiveness of MIBC patients. It is hoped that our results could provide new insights into the field of immunotherapy and references for clinicians to make patient-tailored treatment decisions.

## Materials and methods

### Study population

This study included three independent cohorts. The first cohort enrolled 348 patients from the IMvigor210 trial, a large single-arm phase II study investigating atezolizumab in metastatic urothelial carcinoma (mUC) [7]. All data from the IMvigor210 trial was downloaded from http://research-pub.gene.com/IMvigor210CoreBiologies through the R package *IMvigor210CoreBiologies*. A total of 114 patients were excluded: 50 patients without overall response data and 64 patients without evaluation of TMB level. These patients all had histologically or cytologically documented locally advanced or metastatic urothelial carcinoma (including metastasis from renal pelvis, ureter, urinary bladder, or urethra).

The second cohort contained 412 patients from *the Cancer Genome Atlas* (TCGA) database. Characteristics of the patients and mRNA sequencing data were downloaded from http://www.cbioportal.org/ in July 2021. Twenty-one patients were excluded: 4 patients with non-muscle invasive bladder cancer (NMIBC), 7 patients without survival or sequencing data and 10 patients receiving neoadjuvant chemotherapy. Finally, 391 eligible MIBC patients were included for further survival and bioinformatics analysis. Among them, 335 patients were classified as pure urothelial histology. Fifty-one patients had urothelial carcinoma with variant histology, including 41 squamous, 4 small cell/neuroendocrine, 2 micropapillary, and 4 plasmacytoid. 5 additional tumours were included: 3 pure squamous cell bladder carcinomas, 1 squamous cell carcinoma of non-bladder origin, and 1 bladder adenocarcinoma.

The Zhongshan Hospital (ZSHS) cohort of Fudan University recruited 215 bladder cancer patients treated with radical cystectomy (RC) from 2002 to 2014. Patients with neoadjuvant chemotherapy history were excluded from this cohort. A total of 80 patients were eliminated according to the following exclusion criteria: 60 patients with NMIBC, 13 patients with nonurothelial carcinoma and 7 patients whose specimens detached from the tissue microarray during the process of immunohistochemistry staining. Eventually, the ZSHS cohort was comprised of 135 MIBC patients for further analysis. The pathological type of all these patients was pure urothelial carcinoma. Among them, 65 patients received adjuvant cisplatin-based chemotherapy for at least one therapeutic cycle. The follow-up protocol was instructed by EAU guidelines for MIBC. Overall survival (OS) was calculated as the time from the date of RC to the date of death from all causes, or to the last follow-up. This study was approved by the Clinical Research Ethics Committee of Zhongshan Hospital.

The flow chart of IMvigor210, TCGA and ZSHS cohorts was presented in Supplementary Fig. 1. Baseline characteristics of the three cohorts were summarised respectively in Supplementary Tables 1–3.

### Genomic analysis and variant assessment

TMB was identified as the number of exonic, nonsynonymous single-nucleotide variants (SNV), and indel mutations per megabase of genome examined (mut/Mb) in IMvigor210 and TCGA cohorts. TMB in the TCGA cohort was calculated using results from whole-exome sequencing (WES), which was performed on the Illumina HiSeq platform. TMB in the IMvigor210 trial was obtained by targeted large-panel sequencing named FoundationOne® panel (FMOne). In this study, TMB ≥ 10 mut/Mb was defined as high TMB [6]. We estimated the contributions of mutational signatures (COSMIC v.2, https://cancer.sanger.ac.uk/cosmic/signatures_v2) for each sample in TCGA cohort via “whichSignatures” function of R package *deconstructSigs* (https://bioconductor.org/packages/musicatk/). The data of SNV neoantigens and Indel neoantigens of TCGA cohort was downloaded from previous studies [16].

### RNA-seq data and processing

The RNA-seq data of IMvigor210 and TCGA cohorts were obtained as Fragments Per Kilobase of transcript per Million mapped reads (FPKM) and normalised through the formula log_2_(FPKM + 1) before analyses. The R package *BLCAsubtyping* (https://github.com/cit-bioinfo/BLCAsubtyping) was used to derive the molecular subtype information of each patient. The gene sets of antigen processing and presentation and T cell receptor signalling pathway were derived from *KEGG* database (https://www.kegg.jp/). Gene members of IFNα response, IFNγ response and TGFβ signalling pathway were derived from *Molecular Signatures Database* (MSigDB, http://www.gsea-msigdb.org/). The single sample gene set enrichment analysis (ssGSEA) algorithms were employed to quantify the gene sets involved [17]. The signatures presented in our study (cytotoxic lymphocytes signature, TLS signature, terminally exhausted signature, progenitor exhausted signature, T cell-inflamed GEP) were defined from previous studies and scored as the average expression of related genes [18–21]. The TCR and BCR diversity scores (Shannon Entropy and Richness) of TCGA cohort were downloaded from previous studies [16].

### Immunohistochemistry

Immunohistochemistry (IHC) staining was performed on formalin-fixed, paraffin-embedded tissue microarray (TMA) of ZSHS cohort as described previously [22]. In summary, four groups of double staining were performed: CD68 and HLA-DR to detect M1 macrophages, CD11c and HLA-DR to detect DCs, CD4 and T-bet to detect Th1 cells, CD4 and GATA3 to detect TH2 cells. For the rest of immune cells and immune markers including CD4^+^ T cells (CD4), CD8^+^ T cells (CD8), macrophages (CD68), M2 macrophages (MRC1), Tregs (FOXP3), B cells (CD19), NK cells (CD56), mast cells (mast cell tryptase), neutrophils (CD66b) Th17 cells (IL17A) and PD-L1, we performed single IHC staining. This information was included in Supplementary Table 4. Tissue microarray analyses (TMAs) are from the ZSHS cohort. The existence of intratumoural tertiary lymphoid structures (TLSs) was assessed via both H&E and CD3/CD20 double staining using a previously published scale [23]. Tumours with at least 1 intratumoural TLS were defined as TLS positive (TLS^+^).

In our study, two pathologists (Dr. Lingli Chen and Dr. Yunyi Kong) from different medical institutes who were blinded to the clinicopathological data scored all samples separately. The mean count of their evaluation was adopted. Variations in the enumeration, exceeding 0.15 IHC score or 5 cells, were re-evaluated separately by both pathologists to reach a consensus.

### Construction of the CPT score

In IMvgior210 Trial, PD-L1 expression on tumour-infiltrating immune cells (IC) is a proven biomarker of immunotherapy sensitivity. PD-L1 IC was evaluated by PD-L1 antibody SP142 using the VENTANA platform. Scoring criteria designated tumours as IC0, IC1, or IC2/3 (PD-L1 expression on <1%; ≥1% and <5%; or ≥5% of IC, respectively). Since the data of PD-L1 expressing on tumour-infiltrating immune cells (IC) was not available in TCGA database, we defined the top 40% as high PD-L1 mRNA expression with reference to IMvigor210 cohort (IC2+ accounts for 38.5%) [7]. The cut-off point of PD-L1 in TCGA cohort was 0.7605 FPKM. Consistently, the top 40% was defined as high CD39 expression in the three cohorts involved. The cut-off points of CD39 in IMvigor210 and TCGA cohorts were 7.077 and 3.010 (FPKM), and the cut-off point of CD39^+^ cells in ZSHS cohort was 16 cells/HPF. The CPT score was calculated by integrating three factors (CD39 expression, PD-L1 expression and TMB), which stratified patients into four groups. Score one point for each criterion, including high CD39 expression, PD-L1 expression on ≥5% of tumour-infiltrating immune cells (IC2+) or high PD-L1 expression and TMB ≥ 10 mut/Mb.

### Statistical analyses

The overall survival (OS) was determined by Kaplan–Meier method, which was evaluated by log-rank tests. Univariate and Multivariate Cox regression models were applied to estimate hazard ratios (HRs) and 95% confidence intervals (CIs). Patients’ baseline characteristics and disease factors were presented as descriptive statistics. Results were shown as median and interquartile range (IQR). Mann–Whitney *U*-test, Kruskal–Wallis test, Chi-square test, Fisher’s exact test and Spearman correlation analysis were used in this study. The statistical analyses were performed via IBM SPSS Statistics 25.0 and R4.0.0. Two-sided *P* < 0.05 was regarded as statistically significant.

## Results

### The CPT score identifies patients responsive to PD-L1 blockade in MIBC

**Fig. 1:**
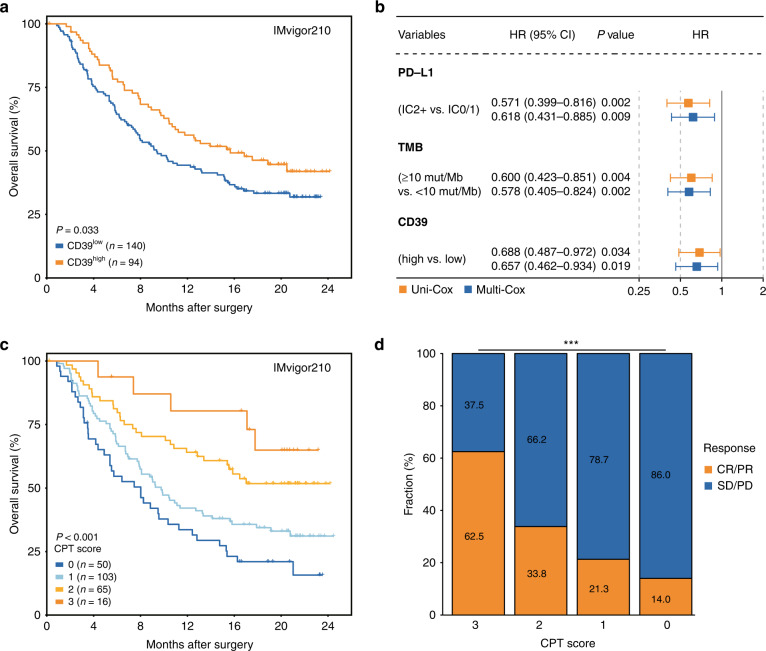
The CPT score based on CD39, PD-L1 and TMB predicts therapeutic efficacy of PD-L1 blockade in MIBC. **a** Kaplan–Meier curve for OS by CD39 expression for patients treated with atezolizumab in IMvigor210 cohort. Log-rank test was performed. **b** PD-L1, TMB and CD39 expression were independent predictive factors for PD-L1 blockade. **c** Kaplan–Meier curve for OS by CPT score for patients treated with atezolizumab in IMvigor210 cohort. Log-rank test was performed. **d** Clinical response to PD-L1 blockade by CPT score in IMvigor210 cohort. Chi-square test was performed (***P < 0.001). OS overall survival, HR hazard ratio, CI confidence interval, PD-L1 programmed death ligand 1, IC0 PD-L1 expression on <1% of tumour-infiltrating immune cells, IC1 PD-L1 expression on 1–<5% of tumour-infiltrating immune cells, IC2+ PD-L1 expression on ≥5% of tumour-infiltrating immune cells, TMB tumour mutation burden, CR complete response, PR partial response, SD stable disease, PD progressive disease.

In the phase II IMvigor210 trial, atezolizumab showed clinical benefit in mUC patients with high PD-L1 expression (≥5%, IC2+) and high TMB [7]. However, 65.5% of IC2+ patients and 58.7% of patients with high TMB could not benefit from atezolizumab (Supplementary Fig. 2A, B). Among the key molecules of the ATP-adenosine pathway, we found that only CD39 expression was significantly correlated with the OS of patients treated with atezolizumab, evaluated with the univariate and multivariate Cox models (Supplementary Fig. 2C). Compared with CD39^low^ subgroup, CD39^high^ subgroup was linked to an inferior OS in IMvigor210 cohort (Fig. 1a, *P* = 0.033). Besides, CD39 expression, PD-L1 expression and TMB all served as independent factors after adjustment of gender, tobacco use history, metastatic site and ECOG PS score, to predict OS of patients from IMvigor210 cohort observed with the multivariate analysis (Fig. 1b and Supplementary Table 5). We further assessed whether CD39 synergising with PD-L1 or TMB could predict response to atezolizumab. Pairwise combination of CD39, PD-L1 and TMB showed improved but unsatisfactory performance in stratification of patients (Supplementary Fig. 2D–F). We then stratified patients into 4 groups based on a “CPT score”, which was constructed by incorporating CD39, PD-L1 and TMB (CPT score = 0, 1, 2, and 3). Remarkably, patients with higher CPT scores showed superior OS after treatment with atezolizumab (Fig. 1c, *P* < 0.001). The AUCs of CPT score for 12-month and 24-month OS were 0.649 and 0.674 respectively, which showed superiority over CD39, PD-L1 or TMB alone (Supplementary Fig. 3). The fractions of patients who achieved complete response (CR) or partial response (PR) were 62.5%, 33.8%, 21.3%, and 14.0% in CPT score = 3, 2, 1, and 0 subgroups, respectively (Fig. 1d, *P* < 0.001).

### The CPT score predicts chemotherapeutic response in MIBC

**Fig. 2:**
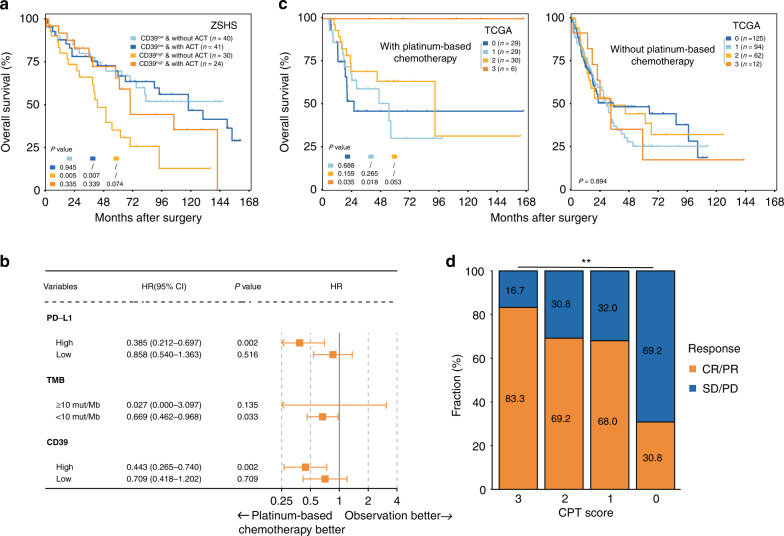
The CPT score predicts chemotherapeutic response in MIBC. **a** Kaplan–Meier analysis for OS stratified by CD39+ cells infiltration and ACT application in ZSHS cohort. Log-rank test was performed. **b** PD-L1, TMB and CD39 expression influenced response to platinum-based chemotherapy in TCGA cohort. **c** Kaplan–Meier analysis demonstrated the overall survival probability stratified by the CPT score in patients with platinum-based chemotherapy or without platinum-based chemotherapy. Log-rank test was performed. **d** Clinical response to platinum-based chemotherapy by CPT score in TCGA cohort. Chi-square test was performed (**P < 0.01). ACT adjuvant chemotherapy.

Cisplatin-based chemotherapy remains the standard systemic treatment for MIBC patients [4]. To explore whether the CPT score was associated with responsiveness to platinum-based chemotherapy, we compared the prognosis of patients stratified by CD39 expression, PD-L1 expression and TMB separately or in combination. Representative images of tumours with low or high CD39^+^ cells infiltration of ZSHS cohort were shown in Supplementary Fig. 4A. CD39^high^ subgroup had significantly inferior OS in ZSHS cohort (Supplementary Fig. 4B, *P* = 0.007). However, the OS of this subgroup could be improved after treatment with adjuvant chemotherapy (ACT) (Fig. 2a). The same result was found in TCGA cohort (Supplementary Fig. 5A). Intriguingly, we also observed that platinum-based chemotherapy could significantly improve the OS of patients with high PD-L1 expression or high TMB in TCGA cohort (Supplementary Fig. 5B, C and Fig. 2b). We also applied “CPT score” in TCGA cohort and found that, notably, patients with the highest CPT score showed the best OS after treatment with platinum-based chemotherapy. While in the group of patients who did not receive platinum-based chemotherapy, there was no statistically significant difference in OS among subgroups with different CPT scores (Fig. 2c). Consistently, patients with a CPT score of 3 manifested the highest response rate (83.3%) to platinum-based chemotherapy (Fig. 2d, *P* < 0.01).

### The CPT score correlates with increased neoantigen burden in MIBC

**Fig. 3:**
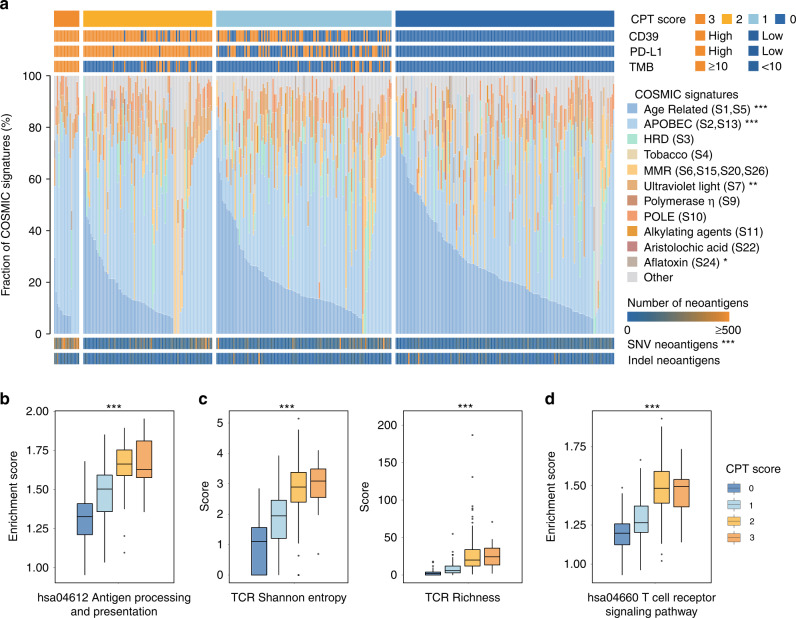
The CPT score correlates with increased neoantigen burden in MIBC. **a** Patterns of mutational signatures and total number of neoantigens in patient subgroups defined by CPT score in TCGA cohort. Kruskal–Wallis test was performed (*P < 0.05; **P < 0.01, ***P < 0.001). **b** The pathway scores of antigen processing and presentation were compared among patient subgroups defined by CPT score. Kruskal–Wallis test was performed (***P < 0.001). **c** Higher CPT score is associated with increased T cell receptor repertoire richness and clonotype diversity. Kruskal–Wallis test was performed (***P < 0.001). **d** The pathway scores of T cell receptor signalling were compared among patient subgroups defined by CPT score. Kruskal–Wallis test was performed (***P < 0.001). HRD homologous recombination deficiency, MMR mismatch repair, SNV single-nucleotide variant, TCR T cell antigen receptor.

Patients with high CPT scores were characterised by dominant APOBEC mutational signatures in TCGA cohort (Fig. 3a). A recent study argued that APOBEC3B-mediated mutations resulted in more neoepitopes in tumour cell vaccines [24]. Consistently, we observed that SNV neoantigens were significantly enriched in patients with higher CPT scores (Fig. 3a). Increased number of copy number segments was observed in patients with the highest CPT score (CPT score = 3) (Supplementary Fig. 6). Furthermore, single-sample gene set enrichment analysis (ssGSEA) revealed that patients with higher CPT scores were characterised by higher level of antigen processing and presentation signature (Fig. 3b, *P* < 0.001). The effective activation of anti-tumour response also relied on the recognition of neoantigens by TCR and BCR repertoires [16]. Compared with patients with low CPT scores, patients with high CPT scores demonstrated a significant increase in TCR and BCR diversity, and downstream T cell receptor signalling pathway (Supplementary Fig. 7A, B and Fig. 3c, d).

### The CPT score represents immunogenic microenvironment in MIBC

**Fig. 4:**
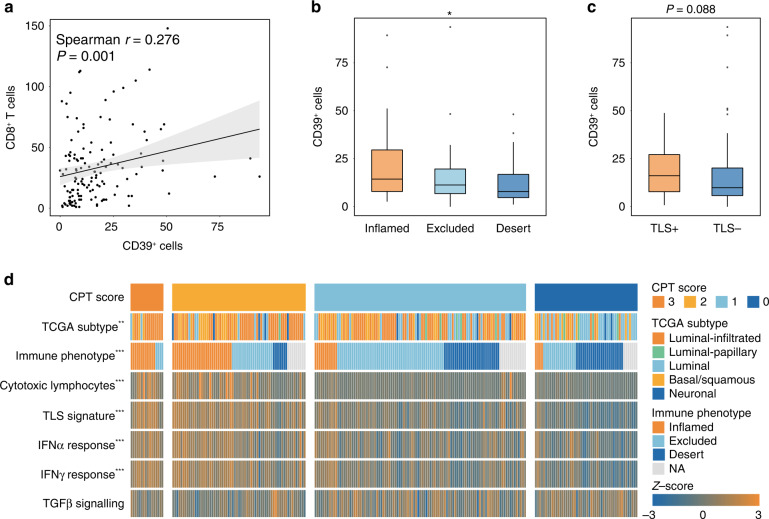
The CPT score represents immunogenic microenvironment in MIBC. **a** Non-parametric two-sided Spearman correlation analysis was performed to figure out the correlations between CD39^+^ cells infiltration and CD8^+^ cells infiltration in ZSHS cohort. **b** Association between CD39^+^ cells infiltration and immunotypes in ZSHS cohort. Kruskal–Wallis test was performed (*P < 0.05). **c** Association between CD39^+^ cells infiltration and tertiary lymphoid structures in ZSHS cohort. Mann–Whitney *U*-test was performed. **d** Heatmap depicting the correlation of CPT score with immune indicators in IMvigor210 cohort. Chi-square test was performed for TCGA subtype and immune phenotype (**P < 0.01; ***P < 0.001). Kruskal–Wallis test was performed for signatures and pathway scores (***P < 0.001). TLS tertiary lymphoid structure.

Previous studies have reported that CD39 was a marker for exhausted T cells, but CD39^+^ T cells displayed effector functions with a great quantity of activation genes expression [12, 25]. We observed that CD39 expression was positively associated with the infiltration of CD8^+^ T cells (Supplementary Fig. 8A and Fig. 4a). Intriguingly, the correlation between CD39 expression and progenitor-depleted T cells signature is stronger than that between terminally depleted T cells signature (Supplementary Fig. 8B, C). Moreover, CD39 expression was significantly linked with inflamed immune contexture (Fig. 4b). TLS^+^ patients were characterised with higher CD39^+^ cells infiltration (Fig. 4c). Together these results suggest that CD39^high^ subgroup exhibited inflamed TME with functional CD8^+^ T cells infiltration.

MIBC patients with high CPT score mainly demonstrated luminal-infiltrated and basal/squamous molecular subtype in IMvigor210 cohort, which were sensitive to immunotherapy [26]. By integrating CPT score and TCGA subtype, we identified that high CPT score indicated better immunotherapeutic efficacy only in patient with luminal-infiltrated and basal/squamous subtypes (Supplementary Fig. 9). High CPT score also indicated the inflamed phenotype, displaying enriched cytotoxic lymphocytes signature and TLS signature (Fig. 4d). We also found that high CPT score indicated an immune-enriched tumour microenvironment in both IMvigor210 and TCGA cohorts (Supplementary Fig. 10). Consistently, patients with high CPT score had a more favourable anti-tumour response with elevated IFNα and IFNγ response (Fig. 4d). Similar results were verified in TCGA cohort (Supplementary Fig. 11).

## Discussion

To optimise patient stratification and therapeutic strategies, multiple biomarkers have been identified to predict immunotherapeutic response, including markers indicative of immune infiltration such as T cell-inflamed gene-expression profile (GEP), and those related to tumour-specific neoantigens such as TMB [8, 21, 27]. In the phase Ib KEYNOTE-028 trial, each of T cell-inflamed GEP, PD-L1 and TMB could predict the clinical efficacy of pembrolizumab individually with only moderate correlation between them [15]. As a key modulator of immune response, the role of CD39 in shaping TME and patient response to systemic therapies has not been fully understood in MIBC so far. In line with previous study, we reported CD39 as an indicator for inflamed TME in MIBC and could serve as a complementary predictor of response to ICIs together with PD-L1 and TMB. Moreover, the “CPT score” constructed based on CD39, PD-L1 and TMB was able to identify patients who were more likely to benefit from ACT.

Over the past two decades, a tremendous number of studies have revealed that efficient elimination of tumour tissues could be achieved via enhancing the immunogenicity of tumours, abrogating immune suppression, and stimulating the activation of effector cells [28]. Previous research has proved that PD-L1 and TMB are important biomarkers for ICIs [29]. However, there are tumours with a high TMB that do not respond. Conversely, there are tumours with low TMB that benefit from immunotherapy. Low spectrum of tumour purity results in low clonally homogeneous TMB, which may not match the actual tumour mutational profile. On the other hand, antigen presentation machinery affected by different mutational signatures may modulate antitumour immune response of different strengths. Platinum-based chemotherapy could enhance tumour-specific neoantigen release via induction of tumour cell apoptosis [30], which might be the reason why patients with high TMB were more sensitive to ACT in our study. However, emerging evidence showed that patients with limited pre-existing immune infiltration failed to benefit from chemotherapy [31]. It has been well established that the expression of PD-L1 indicated a sustained immune response regulated by immunosuppressive factors in TME [32]. Here, we found that the expression of PD-L1 was related to ACT response. Several studies have also observed that PD-L1 expression was correlated with chemotherapy benefit in lung adenocarcinoma and colorectal cancer [33, 34]. Nevertheless, patients with high PD-L1 and TMB may not benefit from ICIs and ACT due to the lack of tumour-specific effector T cells, considering the vast majority of tumour-infiltrating CD8^+^ T cells were bystanders without response to tumour-specific neoantigens [35]. Previous research has established that the success of cancer immunotherapies depends on neoantigen-specific T cell reactivity [36]. We observed higher response rates and longer OS in patients with high level of CD39, which is a marker of tumour-reactive T cells [12]. The correlation between therapeutic benefit and CD39 was also reported in hepatocellular carcinoma and non-small cell lung cancer [37, 38]. Furthermore, considering CD39 expression might represent the existence of tumour-reactive T cells, which was the premise of reactivation, a combination of CD39, PD-L1 and TMB could better stratify patients who received ICIs and ACT. Consistent with the above results, we observed that higher CPT scores were correlated with enriched APOBEC mutational signatures and neoantigens, enhanced antigen presentation and inflamed TME, which have been reported to be indicative of improved immunotherapeutic efficacy [15, 39, 40].

Recently, three antibodies targeting CD39 have entered clinical trials (NCT03884556, NCT04261075, NCT04336098) based on the fundamental insights of downstream immunosuppressive adenosine [41]. Blocking the activity of CD39 can be a promising immunotherapeutic strategy by reducing the synthesis of adenosine and decreasing the fast hydrolysis of pro-inflammatory eATP. In addition, the combination of platinum-based chemotherapy and anti-PD-1/PD-L1 agents has become a research hotspot in the clinic [42]. CD39 expression and the constructed CPT score may also provide guidance for these emerging therapeutic strategies in the future.

There were several limitations in the present study. Our study was retrospective and further confirmation of our findings within the framework of larger and multi-centred clinical trials was required. The thresholds for patient grouping were not bioanalytically validated, which might impact the reproducibility and required further identification of the thresholds. In ZSHS cohort, we did not perform genome sequencing for each patient after surgery since the excessive storage time of the tissue restrict us from further assessing TMB level in these patients at present. The assessment methods of PD-L1 expression and TMB in IMvigor210 and TCGA cohorts were not consistent, which asked for further verification of their correlation.

In summary, the present study demonstrated that CD39 predicted clinical efficacy of PD-L1 blockade or cisplatin-based adjuvant chemotherapy independently in MIBC. A composite of CD39, PD-L1 and TMB showed improved performance in predicting response and could serve as a candidate predictive biomarker for patient-tailored treatment decisions.

## Supplementary information

Supplementary materials

## Data Availability

All data generated that are relevant to the results presented here are included in this article. Other data that were not relevant for the results are available from the corresponding author WZ upon reasonable request.
