# Supplementary material for: Integrative tumour mutation burden with CD39 and PD-L1 for the prediction of response to PD-L1 blockade and adjuvant chemotherapy in muscle-invasive bladder cancer patients
Source: Br J Cancer. 2022 Aug 23;127(9):1718–25. doi: 10.1038/s41416-022-01943-y (PMC9596489; doi:10.1038/s41416-022-01943-y)
Supplement: Supplementary file 1 — Supplementary materials [file 41416_2022_1943_MOESM1_ESM.docx]

**Integrative tumor mutation burden with CD39 and PD-L1 for the prediction of response to PD-L1 blockade and adjuvant chemotherapy in muscle-invasive bladder cancer patients**

**Online-only Supplementary Materials**

**Supplementary Figure 1.** Study flow chart of the three cohorts included in this study.

**Supplementary Figure 2.** Clinical response for PD-L1 blockade by PD-L1, TMB and CD39 expression in IMvigor210 cohort.

**Supplementary Figure 3.** Time-dependent ROC analysis for CPT score, CD39, PD-L1, and TMB in IMvigor210 cohort.

**Supplementary Figure 4.** CD39 expression is associated with significantly shortened overall survival.

**Supplementary Figure 5.** Kaplan–Meier analysis for OS comparing platinum-based chemotherapy to no platinum-based chemotherapy in patient subgroups defined by CD39 expression, PD-L1 or TMB in TCGA cohort.

**Supplementary Figure 6.** Association between CPT score and genomic features in TCGA cohort.

**Supplementary Figure 7.** Higher CPT score is associated with increased B cell receptor repertoire richness and clonotype diversity.

**Supplementary Figure 8.** Association between the immune microenvironment and CD39 expression.

**Supplementary Figure 9.** Prognostic value of CPT score in different molecular subtypes of MIBC.

**Supplementary Figure 10.** Association between CPT score and immune cell subset infiltration.

**Supplementary Figure 11.** Heatmap depicting the correlation of CPT score with immune indicators in TCGA cohort.

**Supplementary Table 1.** Baseline patient characteristics of IMvigor210 cohort.

**Supplementary Table 2.** Baseline patient characteristics of TCGA cohort.

**Supplementary Table 3.** Baseline patient characteristics of ZSHS cohort.

**Supplementary Table 4.** Immunohistochemistry antibodies.

**Supplementary Table 5.** The clinicopathological characteristics influencing overall survival in IMvigor210 cohort analyzed by univariate and multivariate Cox models.


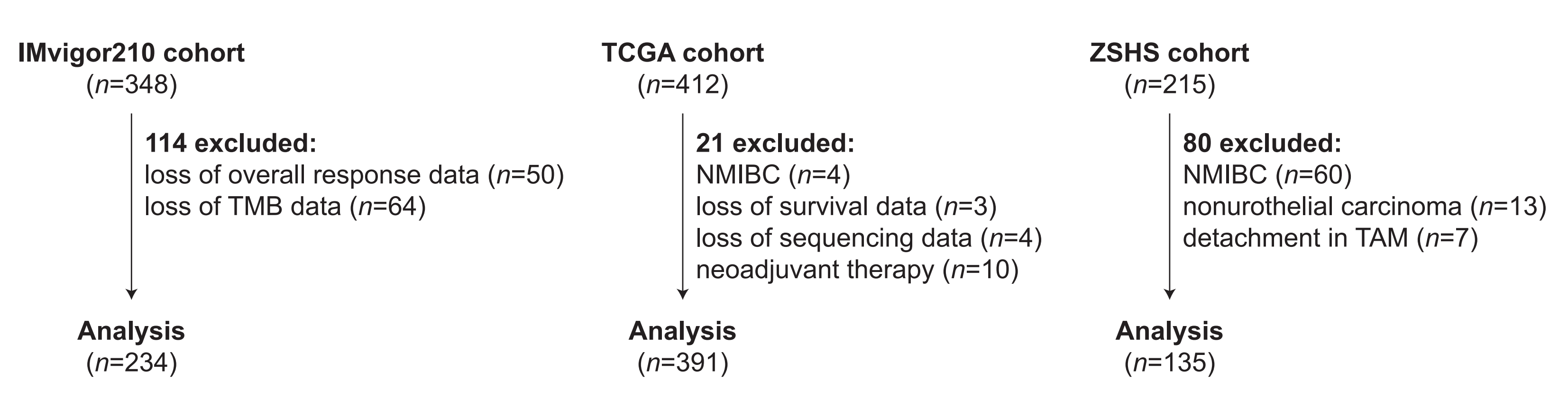


**Supplementary Figure 1. Study flow chart of the three cohorts included in this study.**

**
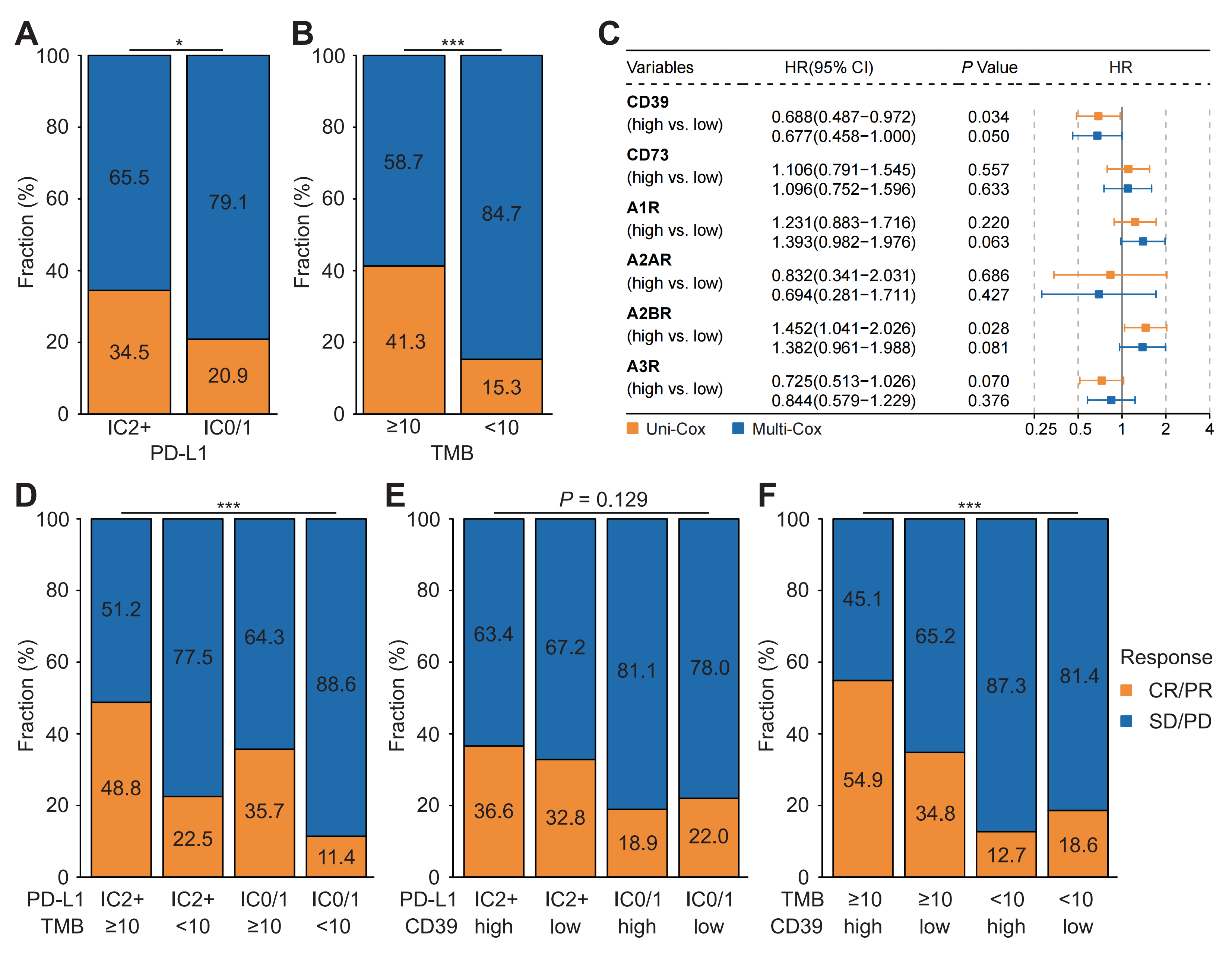
**

**Supplementary Figure 2. Clinical response for PD-L1 blockade by PD-L1, TMB and CD39 expression in IMvigor210 cohort.** **(A-B)** Clinical response to PD-L1 blockade by PD-L1 **(A)** and TMB **(B)**. **(C)** Forest plot showing HRs and 95% CIs for OS in subgroups defined by CD39, CD73 and adenosine receptors expression within the ATP-adenosine pathway. **(D)** Integration of PD-L1 and TMB in patient stratification for response. **(E)** Integration of PD-L1 and CD39 expression in patient stratification for response. **(F)** Integration of TMB and CD39 expression in patient stratification for response. Chi-square test was performed (*, *P* < 0.05; ***, *P* < 0.001).


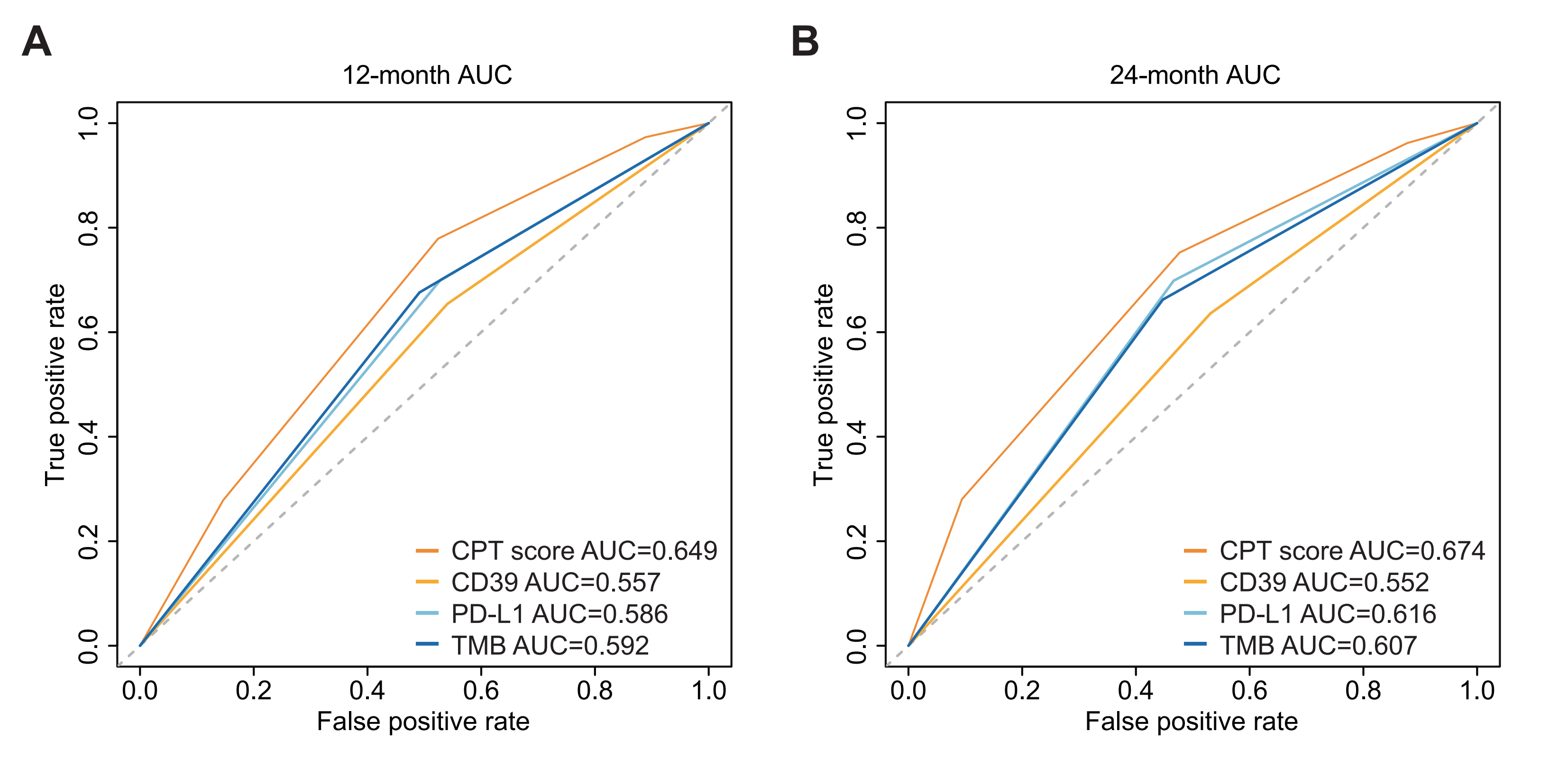
**Supplementary Figure 3. Time-dependent ROC analysis for CPT score, CD39, PD-L1, and TMB in IMvigor210 cohort.** **(A-B)** The time-dependent ROC curves for 12-month **(A)** and 24-month overall survival **(B)**.


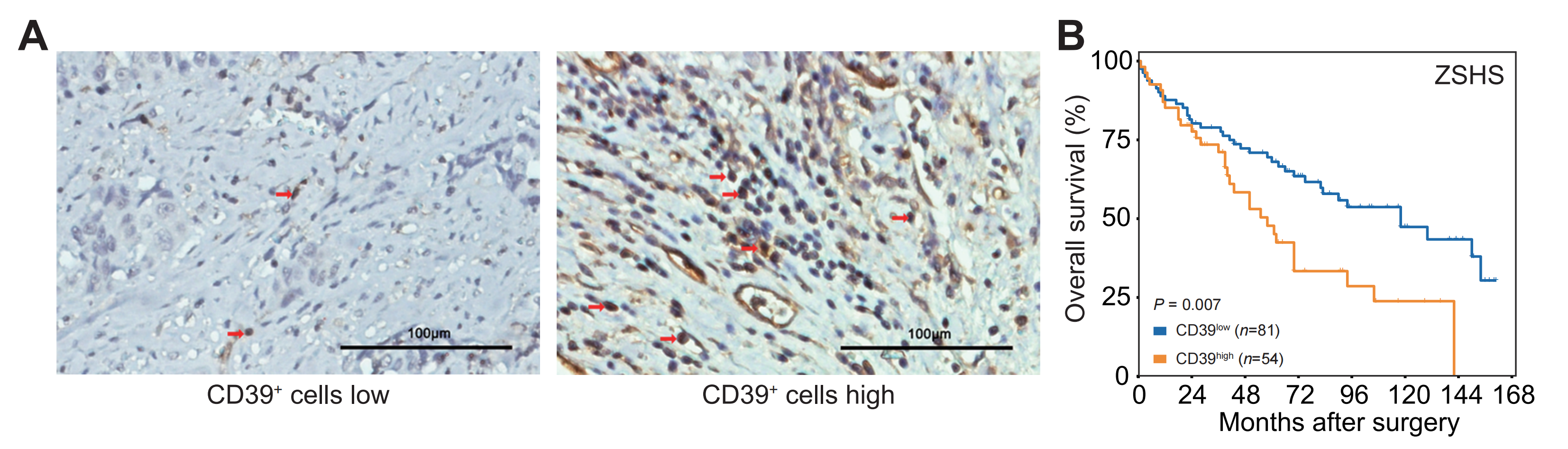


**Supplementary Figure 4. CD39 expression is associated with significantly shortened overall survival.** **(A)** Representative images of tumors with low CD39^+^ cells infiltration (left) and tumors with high CD39^+^ cells infiltration (right). **(B)** Kaplan–Meier curve for OS by CD39 expression for patients in ZSHS cohort. Log-rank test was performed.


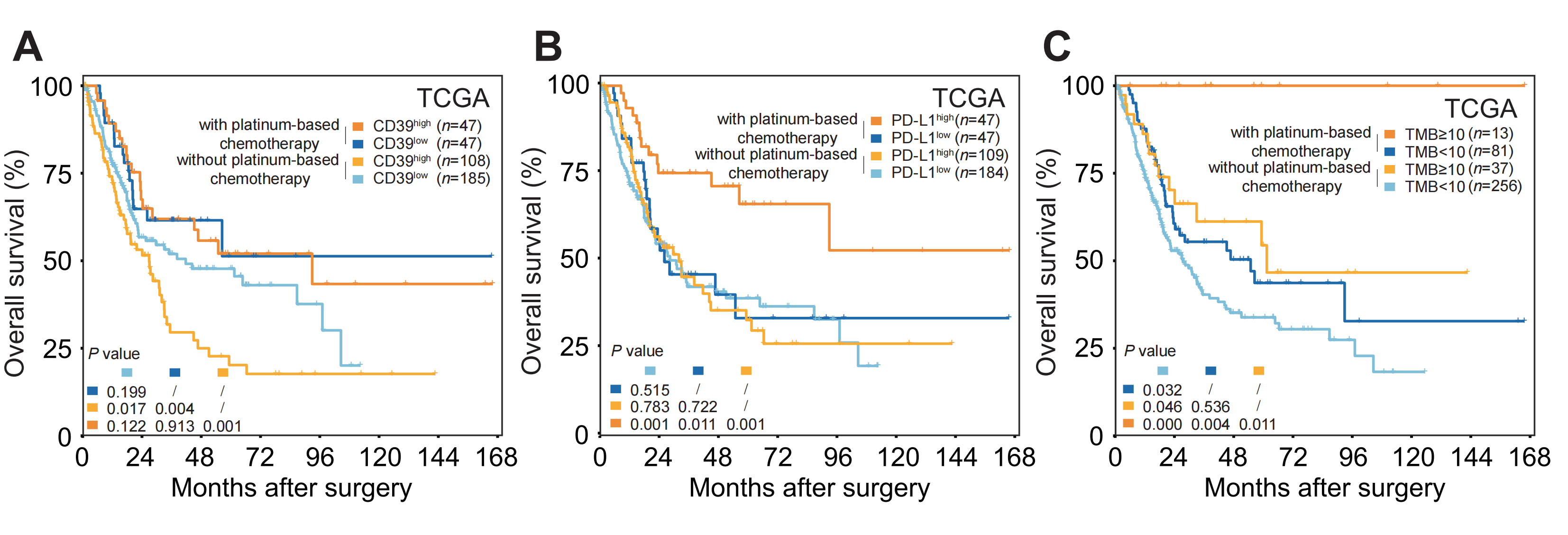


**Supplementary Figure 5. Kaplan–Meier analysis for OS comparing platinum-based chemotherapy to no platinum-based chemotherapy in patient subgroups defined by CD39 expression, PD-L1 or TMB in TCGA cohort.** **(A-C)** CD39 expression **(A)**, PD-L1 **(B)** and TMB **(C)** influenced response to platinum-based chemotherapy. Log-rank test was performed.


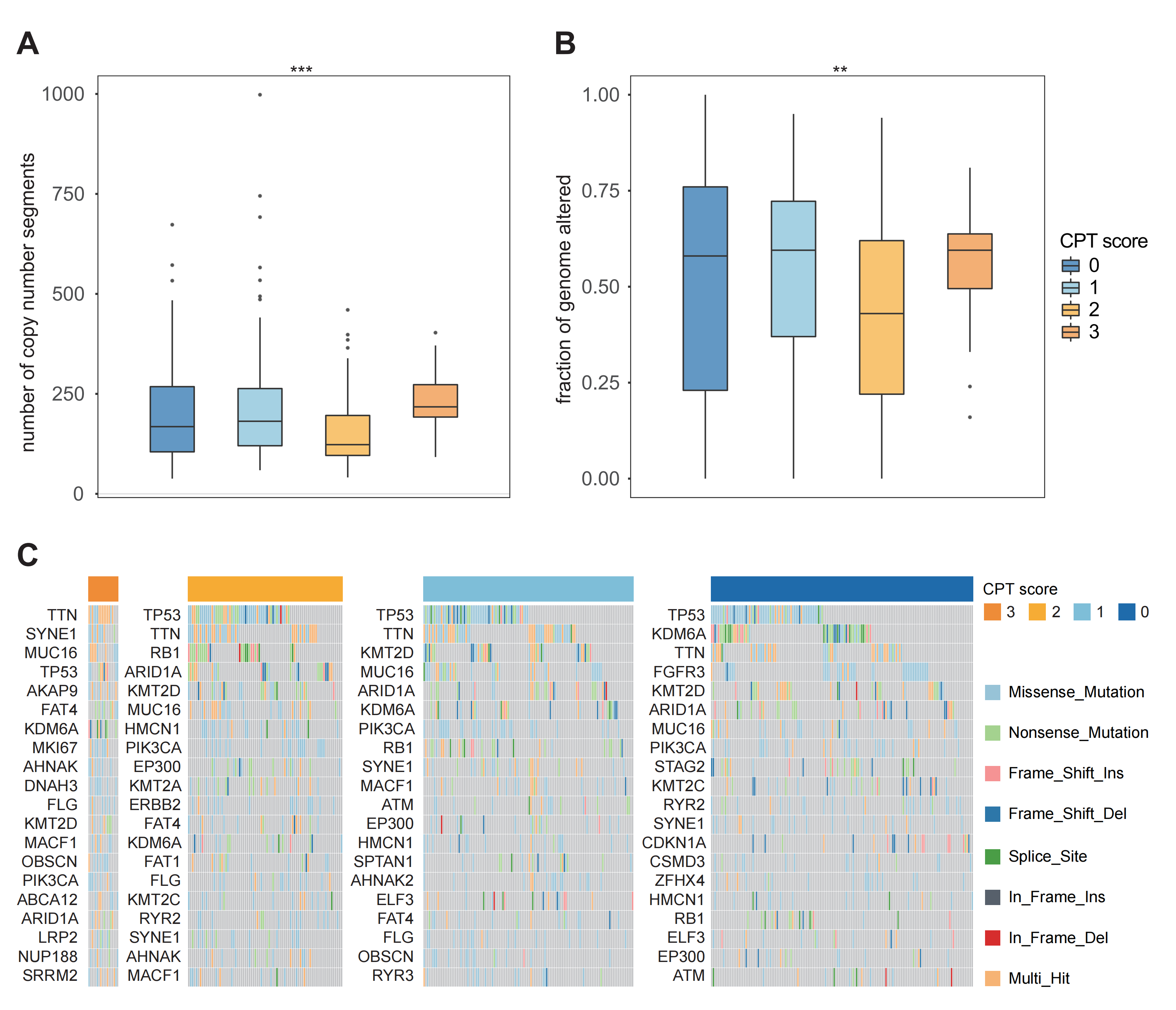
**Supplementary Figure 6.** **Association between CPT score and genomic features in TCGA cohort.** **(A-B)** Number of copy number segments **(A)** and fraction of genome altered **(B)** were used to quantify and compare copy number alternations across subgroups with distinct CPT scores. Kruskal-Wallis test was performed (*, *P* < 0.05; **, *P* < 0.01; ***, *P* < 0.001). **(C)** The landscape of top 20 most frequently mutated genes in patients with varying CPT scores.


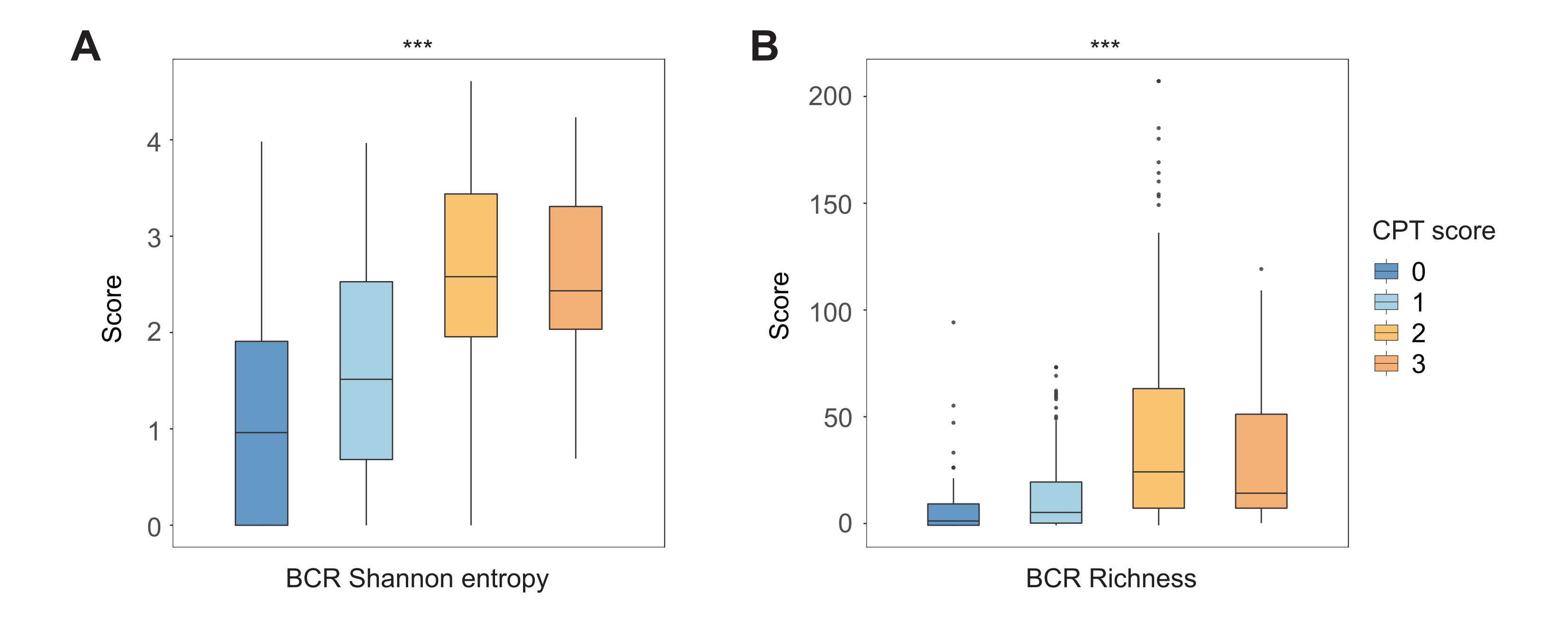


**Supplementary Figure 7. Higher CPT score is associated with increased B cell receptor repertoire richness and clonotype diversity.** **(A-B)** BCR Shannon entropy **(A)** and richness **(B)** were compared among patient subgroups defined by CPT score.


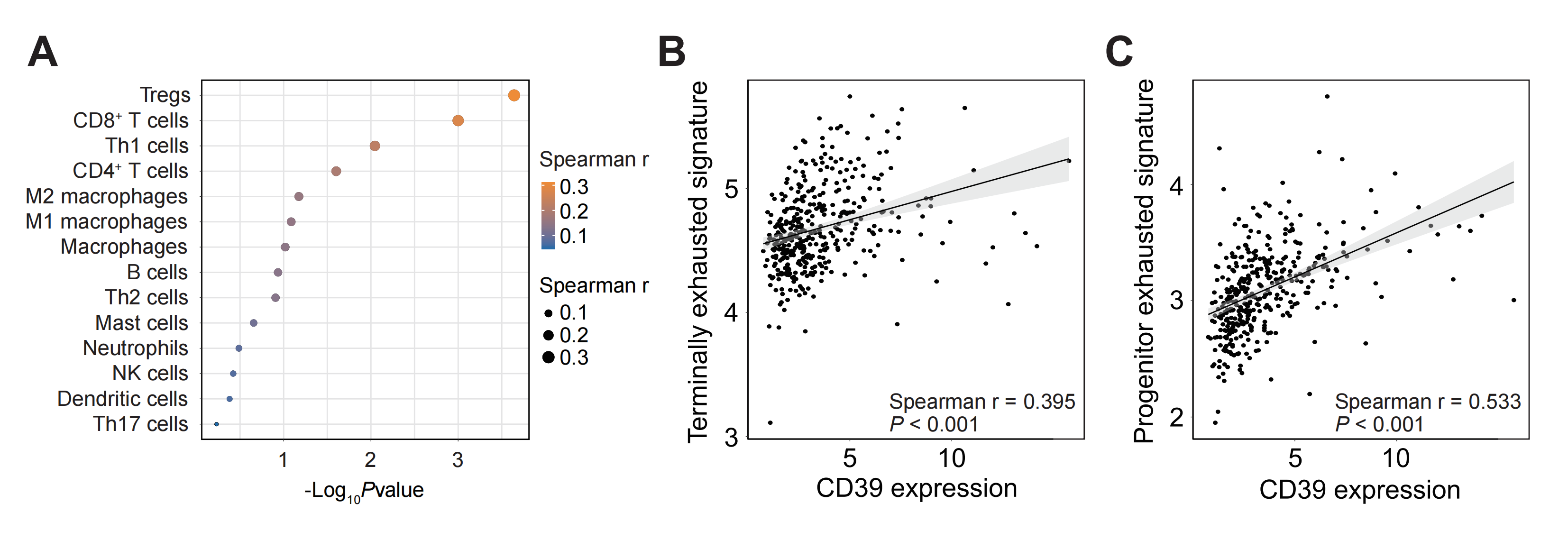


**Supplementary Figure 8. Association between the immune microenvironment and CD39 expression.** **(A)** Association between CD39^+^ cells infiltration and immune contexture in ZSHS cohort. **(B-C)** Non-parametric two-sided Spearman correlation analyses were performed to figure out the correlations between CD39 expression and terminally exhausted **(B)** and progenitor exhausted CD8^+^ T cell signatures **(C)** in TCGA cohort.


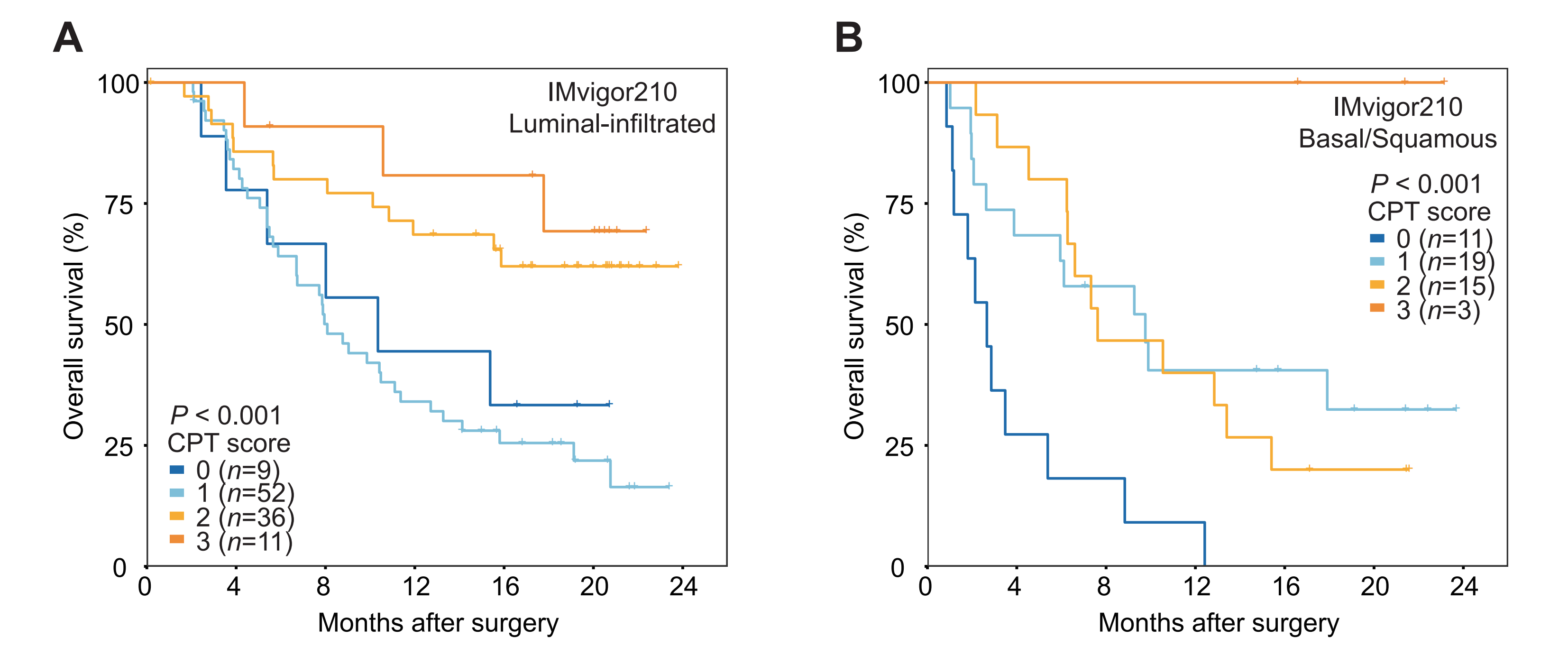
**Supplementary Figure 9. Prognostic value of CPT score in different molecular subtypes of MIBC.** **(A-B)** Kaplan-Meier curves for OS by CPT score in patients with luminal-infiltrated subtype **(A)** and basal/squamous subtype **(B)**. Log-rank test was performed.


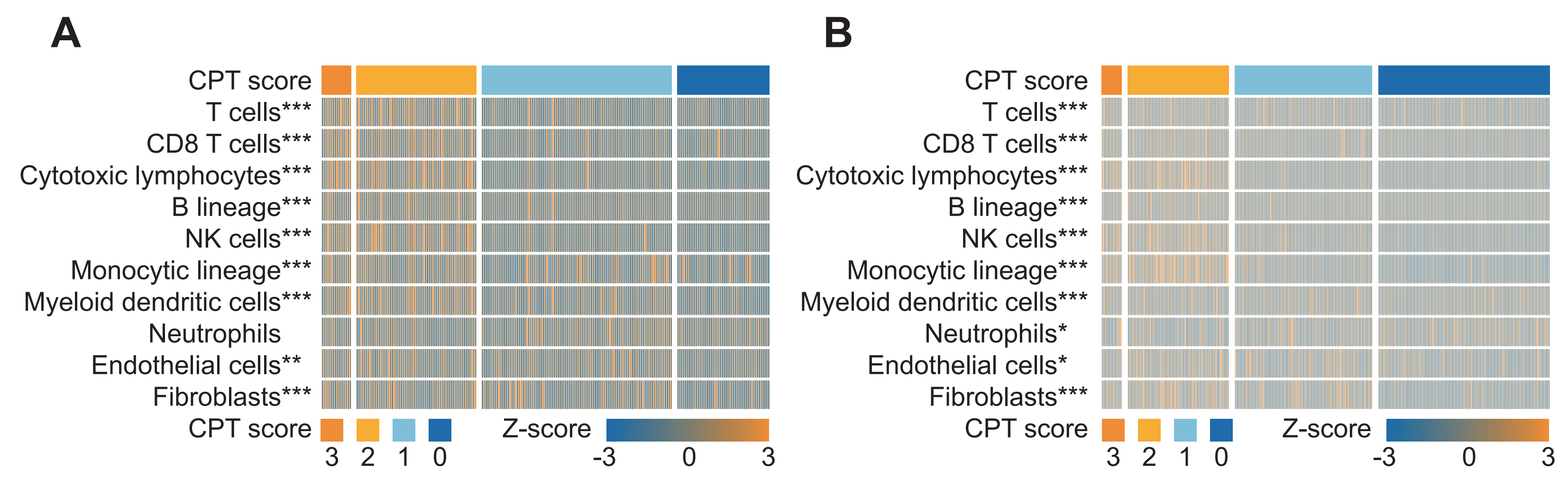
**Supplementary Figure 10. Association between CPT score and immune cell subset infiltration. (A-B)** Varieties of immune cells were highly enriched in subgroups with high CPT scores in both IMvigor210 **(A)** and TCGA **(B)** cohorts. Kruskal-Wallis test was performed (*, *P* < 0.05; **, *P* < 0.01; ***, *P* < 0.001).


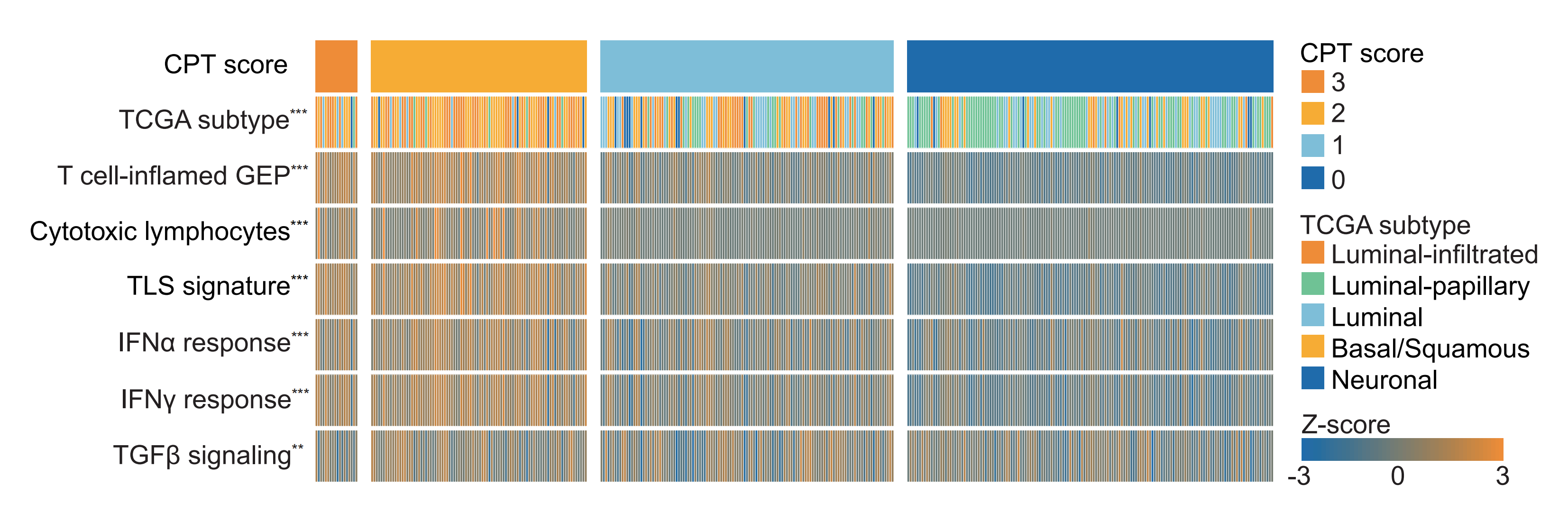


**Supplementary Figure 11. Heatmap depicting the correlation of CPT score with immune indicators in TCGA cohort.**

| **Supplementary Table 1. Baseline patient characteristics of IMvigor210 cohort.** | | | | | | | | |
| --- | --- | --- | --- | --- | --- | --- | --- | --- |
| **IMvigor210 cohort (*n* = 234)** | | | | | | | | |
| **Characteristics** | | **Patients** | | **CPT score** | | | |  |
|  |  | **No.** | **%** | **0** | **1** | **2** | **3** | ***P**** |
| **Total** | | 234 | 100 | 50 | 103 | 65 | 16 |  |
| **Gender** | |  |  |  |  |  |  | 0.655 |
|  | Male | 185 | 79.1 | 37 | 83 | 51 | 14 |  |
|  | Female | 49 | 20.9 | 13 | 20 | 14 | 2 |  |
| **BCG** | |  |  |  |  |  |  | 0.244 |
|  | Applied | 51 | 21.8 | 16 | 21 | 11 | 3 |  |
|  | Not applied | 183 | 78.2 | 34 | 82 | 54 | 13 |  |
| **Platinum-based chemotherapy** | |  |  |  |  |  |  | 0.319 |
|  | Applied | 173 | 73.9 | 36 | 75 | 47 | 15 |  |
|  | Not applied | 61 | 26.1 | 14 | 28 | 18 | 1 |  |
| **TCGA subtype** | |  |  |  |  |  |  | **0.001** |
|  | Luminal-infiltrated | 108 | 46.2 | 9 | 52 | 36 | 11 |  |
|  | Luminal-papillary | 10 | 4.3 | 4 | 5 | 1 | 0 |  |
|  | Luminal | 57 | 24.4 | 24 | 23 | 8 | 2 |  |
|  | Basal/Squamous | 48 | 20.5 | 11 | 19 | 15 | 3 |  |
|  | Neuronal | 11 | 4.7 | 2 | 4 | 5 | 0 |  |
| **Immune phenotype** | |  |  |  |  |  |  | **0.000** |
|  | inflamed | 56 | 27.3 | 4 | 11 | 29 | 12 |  |
|  | excluded | 92 | 44.9 | 16 | 52 | 20 | 4 |  |
|  | desert | 57 | 27.8 | 23 | 27 | 7 | 0 |  |
| **Response** | |  |  |  |  |  |  | **0.000** |
|  | CR/PR | 61 | 26.1 | 7 | 22 | 22 | 10 |  |
|  | SD/PD | 173 | 73.9 | 43 | 81 | 43 | 6 |  |
| **Events** | |  |  |  |  |  |  |  |
|  | Death | 141 | 60.3 | 39 | 67 | 30 | 5 | **0.000** |
|  | Recurrence | / | / | / | / | / | / | / |
| *P** value from Fisher’s exact test was used when data fail to meet the requirement of Chi-square test. | | | | | | | | |
| † Mann-Whitney U test. | | | | | | | | |

| **Supplementary Table 2. Baseline patient characteristics of TCGA cohort.** | | | | | | | | |
| --- | --- | --- | --- | --- | --- | --- | --- | --- |
| **TCGA cohort (*n* = 391)** | | | | | | | | |
| **Characteristics** | | **Patients** | | **CPT score** | | | |  |
|  |  | **No.** | **%** | **0** | **1** | **2** | **3** | ***P**** |
| **Total** | | 391 | 100 | 156 | 125 | 92 | 18 |  |
| **Age at surgery(year)** | |  |  |  |  |  |  | 0.777^†^ |
|  | Median (IQR) | 68(60-76) | | 68(59-76.75) | 70(61-77) | 69(61-75) | 66(60-76.5) |  |
| **Gender** | |  |  |  |  |  |  | 0.512 |
|  | Male | 285 | 72.9 | 117 | 90 | 63 | 15 |  |
|  | Female | 106 | 27.1 | 39 | 35 | 29 | 3 |  |
| **Tumor size (cm)** | |  |  |  |  |  |  | / |
|  | Median (IQR) | / | | / | / | / | / |  |
| **AJCC stage** | |  |  |  |  |  |  | 0.064 |
|  | Ⅱ | 125 | 32.0 | 57 | 36 | 28 | 4 |  |
|  | Ⅲ | 137 | 35.0 | 52 | 36 | 41 | 8 |  |
|  | Ⅳ | 129 | 33.0 | 47 | 53 | 23 | 6 |  |
| **pT stage** | |  |  |  |  |  |  | 0.246 |
|  | pT2 | 113 | 31.6 | 53 | 33 | 25 | 2 |  |
|  | pT3 | 189 | 52.8 | 66 | 62 | 51 | 10 |  |
|  | pT4 | 56 | 15.6 | 20 | 21 | 11 | 4 |  |
| **pN stage** | |  |  |  |  |  |  | **0.004** |
|  | pN0 | 228 | 65.0 | 99 | 56 | 63 | 10 |  |
|  | pN+ | 123 | 35.0 | 41 | 53 | 23 | 6 |  |
| **Grade** | |  |  |  |  |  |  | **0.000*** |
|  | Low grade | 20 | 5.1 | 19 | 1 | 0 | 0 |  |
|  | High grade | 369 | 94.9 | 136 | 124 | 91 | 18 |  |
| **LVI** | |  |  |  |  |  |  | 0.873 |
|  | Absent | 125 | 46.8 | 46 | 43 | 29 | 7 |  |
|  | Present | 142 | 53.2 | 52 | 51 | 34 | 5 |  |
| **ACT** | |  |  |  |  |  |  | 0.079 |
|  | Applied | 94 | 24.3 | 29 | 29 | 30 | 6 |  |
|  | Not applied | 293 | 75.7 | 125 | 94 | 62 | 12 |  |
| **Events** | |  |  |  |  |  |  |  |
|  | Death | 173 | 44.2 | 64 | 64 | 38 | 7 | 0.303 |
|  | Recurrence | 135 | 37.1 | 50 | 54 | 26 | 5 | 0.121 |
| IQR: interquartile range; AJCC: American Joint Committee on Cancer; LVI: lymphovascular invasion; ACT: adjuvant chemotherapy. | | | | | | | | |
| *P** value from Fisher’s exact test was used when data fail to meet the requirement of Chi-square test. | | | | | | | | |
| † Mann-Whitney U test. | | | | | | | | |

| **Supplementary Table 3. Baseline patient characteristics of ZSHS cohort.** | | | | | | |
| --- | --- | --- | --- | --- | --- | --- |
| **ZSHS cohort (*n* = 135)** | | | | | | |
| **Characteristics** | | **Patients** | | **CD39^+^ cells infiltration** | |  |
|  |  | **No.** | **%** | **Low** | **High** | ***P**** |
| **Total** | | 135 | 100 | 81 | 54 |  |
| **Age at surgery(year)** | |  |  |  |  | 0.777^†^ |
|  | Median (IQR) | 62(56-72) | | 62(54-70.5) | 62.5(57.75-72) |  |
| **Gender** | |  |  |  |  | 0.191 |
|  | Male | 112 | 83.0 | 70 | 42 |  |
|  | Female | 23 | 17.0 | 11 | 12 |  |
| **Tumor size (cm)** | |  |  |  |  | 0.638^†^ |
|  | Median (IQR) | 3.5(2.5-5.0) | | 3.5(2.5-5.75) | 3.5(2.5-4.125) |  |
| **AJCC stage** | |  |  |  |  | 0.108 |
|  | Ⅱ | 83 | 61.5 | 45 | 38 |  |
|  | Ⅲ | 44 | 32.6 | 32 | 12 |  |
|  | Ⅳ | 8 | 5.9 | 4 | 4 |  |
| **pT stage** | |  |  |  |  | 0.131 |
|  | pT2 | 86 | 63.7 | 47 | 39 |  |
|  | pT3 | 29 | 21.5 | 22 | 7 |  |
|  | pT4 | 20 | 14.8 | 12 | 8 |  |
| **pN stage** | |  |  |  |  | 0.823 |
|  | pN0 | 127 | 94.1 | 77 | 50 |  |
|  | pN+ | 8 | 5.9 | 4 | 4 |  |
| **Grade** | |  |  |  |  | **0.022** |
|  | Low grade | 22 | 16.3 | 18 | 4 |  |
|  | High grade | 113 | 83.7 | 63 | 50 |  |
| **LVI** | |  |  |  |  | 0.826 |
|  | Absent | 49 | 36.3 | 30 | 19 |  |
|  | Present | 86 | 63.7 | 51 | 35 |  |
| **ACT** | |  |  |  |  | 0.482 |
|  | Applied | 65 | 48.1 | 41 | 24 |  |
|  | Not applied | 70 | 51.9 | 40 | 30 |  |
| **Events** | |  |  |  |  |  |
|  | Death | 70 | 51.9 | 38 | 32 | 0.160 |
|  | Recurrence | 63 | 46.7 | 35 | 28 | 0.324 |
| IQR: interquartile range; AJCC: American Joint Committee on Cancer; LVI: lymphovascular invasion; ACT: adjuvant chemotherapy. | | | | | | |
| *P** value from Fisher’s exact test was used when data fail to meet the requirement of Chi-square test. | | | | | | |
| † Mann-Whitney U test. | | | | | | |

| **Supplementary Table 4. Immunohistochemistry antibodies.** | | | | | | |
| --- | --- | --- | --- | --- | --- | --- |
| **No.** | **Identified cells** | **IHC antibody** | **Company** | **Product No.** | **Clonality Species** | **Diluted** |
| 1 | CD39^+^ cells | Anti-CD39 antibody | Abcam | ab223842 | Monoclonal Rabbit Anti-human | 1:800 |
| 2 | CD4^+^ T cells | Anti-CD4 antibody | Abcam | ab67001 | Monoclonal Mouse Anti-human | 1:50 |
| 3 | CD8^+^ T cells | Anti-CD8 alpha antibody | Abcam | ab17147 | Monoclonal Mouse Anti-human | 1:100 |
| 4 | Macrophages | Anti-CD68 antibody | Abcam | ab955 | Monoclonal Rabbit Anti-human | 1:400 |
| 5 | M1 macrophages | Anti-CD68 antibody | Dako | IR604 | Monoclonal Mouse Anti-human | prediluted |
|  |  | Anti-HLA-DR antibody | Abcam | ab92511 | Monoclonal Rabbit Anti-human | 1:250 |
| 6 | M2 macrophages | Anti-MRC1 antibody | Sigma | HPA004114 | Monoclonal Rabbit Anti-human | 1:500 |
| 7 | Tregs | Anti-FOXP3 antibody | Abcam | ab22510 | Monoclonal Mouse Anti-human | 1:100 |
| 8 | B cells | Anti-CD19 antibody | Abcam | ab31947 | Monoclonal Mouse Anti-human | 1:400 |
| 9 | NK cells | Anti-CD56 antibody | Dako | M7304 | Monoclonal Mouse Anti-human | prediluted |
| 10 | Mast cells | Anti-Mast Cells antibody | Abcam | ab2378 | Monoclonal Mouse Anti-human | 1:10000 |
| 11 | Dendritic cells | Anti-CD11c antibody | Abcam | ab52632 | Monoclonal Rabbit Anti-human | 1:200 |
|  |  | Anti-HLA-DR antibody | Abcam | ab20181 | Monoclonal Mouse Anti-human | 1:200 |
| 12 | Neutrophils | Anti-CD66b antibody | Abcam | ab197678 | Monoclonal Rabbit Anti-human | 1:1000 |
| 13 | Th1 cells | Anti-CD4 antibody | Abcam | ab67001 | Monoclonal Mouse Anti-human | 1:50 |
|  |  | Anti-T-bet antibody | Abcam | ab150440 | Monoclonal Rabbit Anti-human | 1:500 |
| 14 | Th2 cells | Anti-CD4 antibody | Abcam | ab67001 | Monoclonal Mouse Anti-human | 1:50 |
|  |  | Anti-GATA3 antibody | Abcam | ab186371 | Monoclonal Rabbit Anti-human | 1:100 |
| 15 | Th17 cells | Anti-IL17A antibody | Abcam | ab189377 | Monoclonal Mouse Anti-human | 1:1000 |
| 16 | PD-L1 expression | Anti-PD-L1 antibody | Abcam | ab228415 | Monoclonal Rabbit Anti-human | 1:500 |
| 17 | Tertiary lymphoid structures | Anti-CD3 antibody | Abcam | ab16669 | Monoclonal Rabbit Anti-human | 1:75 |
|  |  | Anti-CD20 antibody | Abcam | ab9475 | Monoclonal Mouse Anti-human | 1:100 |

| **Supplementary Table 5. The clinicopathological characteristics influencing overall survival in IMvigor210 cohort analyzed by univariate and multivariate Cox models.** | | | | | |
| --- | --- | --- | --- | --- | --- |
|  | **Univariate Cox** | |  | **Multivariate Cox** | |
| **Characteristics** | **HR (95%CI)** | ***P*** |  | **HR (95%CI)** | ***P*** |

| **Gender** | |  |  |  |  |  |
| --- | --- | --- | --- | --- | --- | --- |
|  | male vs. female | 0.919(0.614-1.735) | 0.681 |  | 0.860(0.566-1.307) | 0.480 |
| **Tobacco use history** | |  |  |  |  |  |
|  | previous/current vs. never | 0.991(0.696-1.413) | 0.962 |  | 0.967(0.669-1.397) | 0.858 |
| **Metastatic site** | |  |  |  |  |  |
|  | visceral vs. lymph node only | 2.286(1.334-3.916) | **0.003** |  | 1.860(1.071-3.231) | **0.028** |
| **ECOG PS** | |  |  |  |  |  |
|  | 2 vs. 0/1 | 1.033(0.707-1.511) | 0.865 |  | 1.199(0.813-1.768) | 0.360 |
| **PD-L1** | |  |  |  |  |  |
|  | IC2+ vs. IC0/1 | 0.571(0.399-0.816) | **0.002** |  | 0.680(0.468-0.988) | **0.043** |
| **TMB** | |  |  |  |  |  |
|  | ≥10 mut/Mb vs. ＜10 mut/Mb | 0.600(0.423-0.851) | **0.004** |  | 0.674(0.465-0.978) | **0.038** |
| **CD39** | |  |  |  |  |  |
|  | high vs. low | 0.688(0.487-0.972) | **0.034** |  | 0.667(0.458-0.970) | **0.034** |
| Visceral metastasis defined as liver, lung, bone, or any nonlymph node or soft tissue metastasis; ECOG PS: Eastern Cooperative Oncology Group performance status. | | | | | | |
